# The Use of Classmates as a Self-Motivation Strategy From the Perspective of Self-Regulated Learning

**DOI:** 10.3389/fpsyg.2019.01314

**Published:** 2019-06-04

**Authors:** José Manuel Suárez, Ana Patricia Fernández, Ángela Zamora

**Affiliations:** ^1^Departamento MIDE II, Universidad Nacional de Educación a Distancia (UNED), Madrid, Spain; ^2^Departamento de Pedagogía, Universidad de Valladolid, Valladolid, Spain

**Keywords:** self-regulated learning, self-motivational strategies, academic motivation, gender, goal orientations

## Abstract

It can be stated that self-regulated learning (SRL) brings broad benefits to the process of students’ learning and studying. However, research has yet to be undertaken in relation to one of its components, namely self-regulation of motivation and affectivity. The main objectives of this study are to examine the use of self-motivation strategies that involve classmates and to obtain models on the influence of academic goals and self-efficacy on such self-motivation strategies. To this end, was conducted a study using two different samples of students in the compulsory secondary education or baccalaureate stages in Spain (*N* = 613 and *N* = 910). The results obtained indicate that, with regard to gender, differences only exist in the use of the strategy of deception (*t* = 5.450, *p* < 0.001, *d* = 0.364). That the two pairs of strategies positively and significantly correlated with one another (*r* = 0.239, *p* < 0.01 and *r* = 0.355, *p* < 0.01). That only the strategy of annulation of others correlates with a more adaptive type of motivation. Thus, the group of students that reported the greatest level in its use also did so in relation to task and ego self-enhancing goals, to self-efficacy, and being negatively associated with the goal of work avoidance. Finally, were offered models on relationships between academic goals, self-efficacy and enhancement and annulation strategies [χ^2^(8) = 5.204, *p* = 0.736] and deception and annulation strategies [χ^2^(4) = 3.228, *p* = 0.520].

## Introduction

The concept of self-regulated learning (SRL) has been defined as the degree to which students are metacognitive, motivational and behaviorally active with regard to their own learning process (Zimmerman, 2013). However, a large proportion of learning now takes place interactively (Järvenoja et al., 2015). For this reason, this article is focused on the study of processes in which the student uses their classmates as a reference point for their own academic motivation. It addresses the self-regulation of the student’s own motivation when they undertake specific evaluations and comparisons regarding the abilities of fellow students within a social context.

It can be said that the broad benefits of SRL have been proved (e.g., Pintrich and De Groot, 1990; Zimmerman, 2013; Järvenoja et al., 2015). Considering SRL offers several benefits to students’ learning and products. For example, studying for exams, developing creative ideas, problem solving, long-term retention, motor tasks and skills that can be learned (Ariel and Karpicke, 2018; Callan et al., 2019), and in general with problems that are emerging in the educational world at present. Nevertheless, it requires that teachers show students how to use these techniques and students will adopt them. Thus, increasing understanding of SRL would inform educators of SRL procedures (Callan and Cleary, 2018).

However, research has yet to be undertaken in relation to some of the most basic components of SRL. While its behavioral and cognitive components have received significant interest, the study of the motivational component seems to have prioritized a focus on the study of motivation rather than on an examination of self-regulation of motivation. This is the case in spite of the fact that strategies of motivational self-regulation or of self-motivation can play an important role in the learning process, as they are aimed at generating and managing various motives and emotions (e.g., Pekrun et al., 2002; Scholer et al., 2018) which are prerequisites for initiating, directing, and maintaining behaviors.

Motivational self-regulation strategies have been addressed through a series of studies in which strategies such as self-handicapping, defensive pessimism and self-affirmation are considered (e.g., Kathryn and Maureen, 2010; Clarke and MacCann, 2016). Nevertheless, these studies do not deal with strategies that, based on a more situational approach, have classmates as a point of reference that can active the use of self-motivation strategies.

Wolters’s (1998) attempt to collectively study various motivational self-regulation strategies gave rise to a subsequent suggesting of six strategies (Wolters and Benzon, 2013). However, there was no incorporation within that broader approach of strategies that, based on a more situational perspective, use classmates as a self-motivation strategy. Suárez and Fernández (2011a), meanwhile, have attempted to provide a more complete approach to motivational self-regulation. To this end, their departure point was Pintrich and De Groot’s (1990) motivational approach. Thus, they used the three motivational components of Pintrich and De Groot’s (1990) to incorporate particular types of self-motivational strategies, putting forward three components of motivational self-regulation strategies: expectations, worth and affectivity. The present studies are focused on the strategies of enhancement of others, annulation of others, deception, and annulation others, which Suárez and Fernández (2011a) located within the strategic components of expectatives and affect.

Accordingly, based on the study of particular self-motivation strategies, the consideration of approaches that offer a more structured view of the different types of these strategies, and ultimately the incorporation of strategies that via a more situational approach use classmates as a self-motivation strategy, it is possible to appreciate how the initial approach of this work has been constructed. Therefore, in this article, we will first of all study the strategies of enhancement and annulation of others. A second part of the study will deal with the strategies of deception and comparison.

Through the strategy of enhancement of others, the student tries to protect their image, attributing their poor results not to low ability or effort on their part or to bad luck, but rather to their peers’ very high capability in academic terms (or at least that of some of them). The student enhances their peers’ qualities, though this does not necessarily have to be associated with a sense of admiration. Rather, it can even lead to a certain resentment or discomfort in relation to them.

Although in the case of enhancement of others the student enhances their peers’ abilities, in the strategy of annulation they do the exact opposite – that is, they deny or minimize their peers’ abilities. This can be achieved in different ways, such as not appreciating the qualities, contributions and good results of their peers or minimizing or simply ignoring them. Through doing so, the student consciously or unconsciously aims to avoid evidence that would allow a comparison in which their image came off worse, thereby letting them continue to see themselves as a student who is no less competent.

The self-motivational strategy of comparison is a characteristic of students who prioritize the pursuit of academic performance as a goal rather than the objective of learning, and for whom the reference point for determining that performance does not comprise the student’s own results only, but also, and even primarily, comparison with the performance of students and peers in their environment. For this reason, the student uses comparison with others when their performance is superior to that of the students relative to whom they are making a comparison, in order to thereby derive feelings of satisfaction, worth and pride. On the other hand, they will seek to avoid this comparison when their performance is lower so as to avoid feelings of shame, guilt, anger or frustration.

Similarly to the comparison strategy, the deception strategy is exhibited by students whose primary goal is performance and whose main reference point is not their own performance, but comparison with the performance of students or peers in their immediate environment. For this reason, the student constructs the expectation of not being surpassed by others through various forms of deception in relation to their peers, such as lying about work carried out for exams, subjects and assignments or attributing any good exam results on their part to external factors.

It has been observed that the strategies of enhancement or annulation of others are related to and influence the development of cognitive strategies but not metacognitive strategies (Suárez and Fernández, 2011b), with the same occurring in the case of deception and comparison strategies (Suárez and Fernández, 2013). However, there are no studies on how such self-motivation strategies are related to students’ affective-motivational variables. The role played by academic goals must be closely considered to this end, because goals are considered as the closest regulators of behaviors. Accordingly, SRL is defined as the regulation of learning that the student develops in order to achieve their personal goals (Nietfeld et al., 2014). A more innovative contribution has been the consideration given to the possibility that several goals simultaneously operate within students (e.g., Hyunjoo, 2012). The different types of goals were considered to simultaneously interact and influence the learning process. For this reason, this approach of multiple goals is also keep in mind in the present work. Another motivational variable that should be taken into account is self-efficacy, which may be described as the belief in one’s own ability to organize and carry out the actions necessary to achieve specific goals (Bandura, 1997).

Self-regulated learning is a key competency that students should learn (Steinbach and Stoeger, 2018). Increasing understanding of SRL would inform educators of SRL procedures (Callan and Cleary, 2018) in order to show their students to use these techniques and thus to impact on students’ learning and achievement. With the work that is presented here, was sought to fill in the gap that exists in research regarding motivational self-regulation strategies based on students’ use of evaluations and comparisons with their classmates. Our research question was if enhancement of others, annulation of others, deception, and comparison strategies can be explained by academic goals and self-efficacy. Thus, our main objectives are firstly to study the use of these strategies, and secondly, to obtain initial exploratory models for each of these relating them to the academic goals and control and self-efficacy beliefs. It was also intended to verify if such models are appropriate regardless of gender, since the literature describes broad differences between the genders in terms of both academic motivation and the use of strategies (e.g., King, 2016; Suárez et al., 2016), as well as in relation to students’ profiles in terms of the adoption of multiple goals. We expected low or middle use of the motivational self-regulatory strategies and that these motivational self-regulatory strategies would be explained by the motivational variables but not regardless of gender and motivational profile.

## Materials and Methods

### Participants and Procedure

The participants in this first sample were a total of 613 students located in Spain, from different contexts, who were in the third year (46.9%) and the fourth year (37.5%) of compulsory secondary education, and the first year (7.3%) and the second year (8.2%) of baccalaureate studies. Of the students, 41.4% are boys and 57.1% are girls. Their average age was 15.69 (SD = 1.271) years.

The participants in second sample were a total of 910 students located in Spain, from different contexts, who were in the third year (36.9%) and the fourth year of compulsory secondary education (39.2%), and in the first year (16.0%) and the second year (7.8%) of baccalaureate studies. Of the students, 46.5% are boys and 52.7% are girls. Their average age was 15.82 (SD = 1.699) years. Learning or development disorders were not informed in both samples.

Non-probability sampling techniques were employed in order to select the study participants. Students were assured that their responses were confidential and that only the researchers would have access to the data. They were told that this was not a test, so they completed the instruments anonymously, with pencil and paper. Finally, they were also informed that there were no right or wrong answers, but only statements that reflect their attitudes and behaviors during learning and studying. This study was carried out in accordance with the Declaration of Helsinki and ethical guidelines. Procedures followed were approved by the Research Ethics Committee of the UNED that waived the need for written informed parental consent to be obtained. The informed consent of the participants was implied through survey completion.

### Variables and Instruments

For data collection, this study used a questionnaire focused on studying various relevant variables. Through it, was collected information on academic goals, beliefs on control and self-efficacy for learning, performance self-efficacy and self-motivation strategies. The instrument items were answered on a five-point scale from 1 (strongly disagree) to 5 (strongly agree).

To devise the part of the questionnaire that was used to measure academic goals, was used Skaalvik’s (1997) Academic Goals Questionnaire. This questionnaire integrated four goal orientations, which were: task, ego self-enhancing, ego self-defeating, and work avoidance goals.

To measure performance self-efficacy and beliefs on control and self-efficacy for learning, was used the corresponding items of the Motivated Strategies for Learning Questionnaire (MSLQ) by Pintrich et al. (1991).

Finally, to assess self-motivation strategies were used the corresponding items from the Scale of Motivational Strategies for Learning-Secondary Version by Suárez and Fernández (2011a). More specifically, with the first sample were evaluated the self-motivational strategies of enhancement of others and of annulation of others. And with the second sample were used the strategies of deception and comparison.

The Spanish version of the instruments have evidence of validity in previous research (e.g., Suárez and Fernández, 2011b, 2013; Suárez et al., 2016; Torrano and Soria, 2017; Suárez and Suárez, 2019).

### Data Analysis

The analysis involved the application of a series of descriptive analyses and correlations. These tests were applied to the different types of academic goals, performance self-efficacy, beliefs on control and self-efficacy for learning and the self-motivation strategies of enhancement and annulation of others. Then focused on gender and clustering of students according to their motivation in subsequent analyses. To this end, were carried out a multivariate analysis of variance (MANOVA) in order to verify the existence of differences based on gender. These differences were also evaluated by means of partial eta square to estimate effect size (η_p_^2^). Then checked if different clusters of students, using Quick Cluster Analysis, could be obtained on the basis of their motivation. Analyses were performed using SPSS 25.0 version. Excel 14.0 was used to calculate Average Variance Extracted (AVE), Composite Reliability (CR), and McDonald’s Omega (OM).

Finally, was used the technique of structural equation modeling using AMOS 22 (Arbuckle, 2013), with the aim of putting forward an explanatory model of how the self-motivational strategies of enhancement and annulation are affected by the motivational variables studied. Structural equation modeling allows three strategies to be adopted. To obtain the model that was set out in this study and to avoid a completely exploratory approach, was adopted the strategy of model development. The estimation technique used was maximum likelihood and the estimation process used was direct estimation. Simultaneous analyses of several groups were performed to fit a model to several sets of data at once (by genders and clusters). In this way, simultaneous analysis of groups provides more accurate parameter estimates than would be obtained from separate single-group analyses (Arbuckle, 2013).

## Results

### Descriptive and Correlational Study

The results of the descriptive and correlational analysis can be found in Tables 1, 2. We observed with the first sample (see Table 1) that the two self-motivation strategies correlate significantly and positively with one another and that both strategies correlate significantly and positively with the two ego-orientation goals. However, the strategy of praising others, unlike the strategy of denigrating others, correlates negatively with the task goal and with control and self-efficacy beliefs, while it correlates positively with the work-avoidance goal.

**Table 1:** Means, standard deviations, and correlations between variables.

	*M*	SD	α	AVE	CR	OM	2	3	4	5	6	7	8
Task (1)	3.874	0.807	0.82	0.49	0.78	0.78	0.259**	0.147**	-0.235**	0.452**	0.288**	-0.116**	0.062
Self-enhancing (2)	2.841	0.946	0.85	0.54	0.82	0.82		0.196**	-0.053	0.148**	0.167**	0.302**	0.220**
Self-defeating (3)	2.734	1.144	0.87	0.64	0.87	0.87			0.050	-0.028	0.016	0.254**	0.069*
Work-avoidance (4)	2.867	0.905	0.69	0.43	0.75	0.75				-0.108**	-0.166**	0.180**	0.009
Control and self-efficacy for learning (5)	3.672	0.766	0.86	0.47	0.76	0.76					0.317**	-0.228**	0.042
Performance self-efficacy (6)	3.133	1.049	0.91	0.46	0.75	0.75						-0.078*	0.119**
Enhancement (7)	1.976	0.902	0.71	0.51	0.81	0.81							0.239**
Annulation (8)	2.560	0.967	0.68	0.42	0.66	0.66							

^∗∗^*p < 0.01; ^∗^*p* < 0.05. AVE, Average Variance Extracted; CR, Composite Reliability; OM, McDonald’s Omega*.

**Table 2:** Means, standard deviations, and correlations between variables.

	*M*	SD	α	AVE	CR	MO	2	3	4	5	6	7	8
Task (1)	3.965	0.725	0.82	0.46	0.77	0.77	0.191**	0.103**	-0.258**	0.386**	0.198**	-0.099**	0.043
Self-enhancing (2)	2.758	0.996	0.85	0.54	0.83	0.83		0.215**	0.055	0.116**	0.202**	0.287**	0.338**
Self-defeating (3)	2.635	1.158	0.87	0.66	0.88	0.88			0.029	-0.102**	-0.017	0.226**	0.320**
Work-avoidance (4)	2.895	0.953	0.69	0.46	0.77	0.77				-0.133**	-0.213**	0.171**	0.123**
Control and self-efficacy for learning (5)	3.700	0.788	0.86	0.48	0.78	0.78					0.325**	-0.107**	-0.154**
Performance self-efficacy (6)	3.091	1.119	0.91	0.49	0.79	0.79						0.052	-0.100**
Deception (7)	1.818	0.717	0.69	0.33	0.60	0.60							0.355**
Comparison (8)	2.226	0.930	0.71	0.28	0.53	0.53							

^∗∗^*p < 0.01. AVE, Average Variance Extracted; CR, Composite Reliability; OM, McDonald’s Omega*.

With respect to the second sample, it was observed that the two strategies of self-motivation correlated significantly and positively with one another; that both strategies correlated significantly and positively with the two goals of ego orientation and with the work-avoidance goal; and that both correlated significantly and negatively with control and self-efficacy for learning. In addition, the deception strategy correlates negatively with the task goal, while the comparison strategy does so with performance self-efficacy.

### Consideration of Gender and Groups of Students With Different Motivational Characteristics

With a view to studying the appropriateness of considering gender, were conducted means difference analysis in relation to the rest of the variables studied. The results of this analysis suggest that the goals of ego self-defeating and work avoidance presented statistically significant differences according to gender (Table 3). Girls scored more highly in the ego self-defeating goal, whereas boys scored more highly in the work-avoidance goal.

**Table 3:** Means, standard deviations, and differences by gender (sample 1 and sample 2).

Strategy	Gender	*M*	SD	*F*	*p*	η_p_^2^
Task	Male	3.811	0.839	3.449	0.064	0.006
	Female	3.925	0.769			
Self-enhancing	Male	2.915	0.909	2.546	0.111	0.004
	Female	2.773	0.961			
Self-defeating	Male	2.563	1.039	13.711	0.000	0.022
	Female	2.866	1.203			
Work-avoidance	Male	3.002	0.844	10.849	0.001	0.018
	Female	2.763	0.937			
Control and self-efficacy for learning	Male	3.669 3.686	0.820 0.730	0.079	0.779	0.000
	Female					
Performance self-efficacy	Male	3.101	1.050	0.299	0.585	0.000
	Female	3.149	1.058			
Enhancement	Male	2.055	0.909	1.478	0.225	0.002
	Female	1.922	0.890			
Annulation	Male	2.620	0.939	0.995	0.319	0.002
	Female	2.532	0.986			
Task	Male	3.899	0.747	6.215	0.013	0.007
	Female	4.019	0.702			
Self-enhancing	Male	2.949	1.007	30.480	0.000	0.033
	Female	2.590	0.950			
Self-defeating	Male	2.492	1.109	13.194	0.000	0.014
	Female	2.771	1.189			
Work-avoidance	Male	3.076	0.965	26.998	0.000	0.029
	Female	2.751	0.912			
Control and self-efficacy for learning	Male	3.694	0.814	0.021	0.886	0.000
	Female	3.702	0.769			
Performance self-efficacy	Male	3.069	1.084	0.253	0.783	0.000
	Female	3.107	1.153			
Deception	Male	1.952	0.732	30.003	0.000	0.032
	Female	1.695	0.676			
Comparison	Male	2.233	0.920	0.078	0.781	0.000
	Female	2.216	0.939			

In line with consideration of students’ motivational characteristics, especially with regard to the adoption of multiple goals, and applying cluster analysis to the data collected, was observed that a clear grouping of students into three groups with different motivational characteristics (Table 4) was obtained. Moreover, this clustering is associated with a different use of strategies of enhancement and annulation of others. The students from the first group are those who reported the highest score in task and ego self-praise goals, in control and self-efficacy for learning, in performance self-efficacy and in the strategy of denigrating others, whereas, on an opposite basis due to its content, it is the group that obtained the lowest score for the goal of work avoidance. The second cluster comprises students who reported higher scores in ego self-frustration and work-avoidance goals and in the strategy of praising others. And, finally, the third cluster comprises students who reported the lowest scores in all variables, except in the work-avoidance goal.

**Table 4:** Centers of clusters by motivational and self-motivation variables (sample 1 and sample 2).

	Cluster		
	1	2	3
Task	4.37	3.80	3.48
Self-enhancing	3.18	3.11	2.16
Self-defeating	2.52	3.57	1.89
Work-avoidance	2.37	3.11	3.05
Control and self-efficacy for learning	4.16	3.48	3.45
Performance self-efficacy	3.98	2.82	2.66
Enhancement	1.59	2.62	1.51
Annulation	2.92	2.67	2.04
Students in cluster	190	240	183

	**Cluster**		
	**1**	**2**	**3**

Task	3.70	4.33	3.85
Self-enhancing	2.09	3.16	3.04
Self-defeating	1.76	2.53	3.70
Work-avoidance	3.01	2.47	3.24
Control and self-efficacy for learning	3.55	4.15	3.37
Performance self-efficacy	2.48	4.07	2.68
Deception	1.53	1.77	2.18
Comparison	1.77	2.08	2.88
Students in cluster	311	314	285

With respect to the second sample, the results of means difference analysis suggest that the four goals and deception strategy present statistically significant differences according to students’ gender (Table 3). Boys showed higher levels in the goals of self-enhancing and work avoidance and in the strategy of deception. Girls showed higher levels in task and ego self-defeating goals. With regard to the rest of the variables, the differences found are not statistically significant.

By applying cluster analysis was observed a clear clustering of students in three groups with different motivational characteristics (Table 4). Moreover, this clustering is associated with a different use of strategies of deception and comparison. In the first cluster students present the lowest levels in task goal and in the two goals oriented to the ego. And they also exhibit the lowest levels in the use of the two self-motivation strategies. The second clustering exhibits the highest task and self-enhancing goals, as well as the lowest level for the work-avoidance goal. And finally, a third cluster comprises students showing higher levels in self-defeating and work avoidance goals. And they also report the greatest use of the two strategies of deception and comparison.

### A Model About the Relationship of the Strategies of Enhancement and Annulation of Others, Considering Gender and Groups With Different Motivational Characteristics

**Figure 1:**
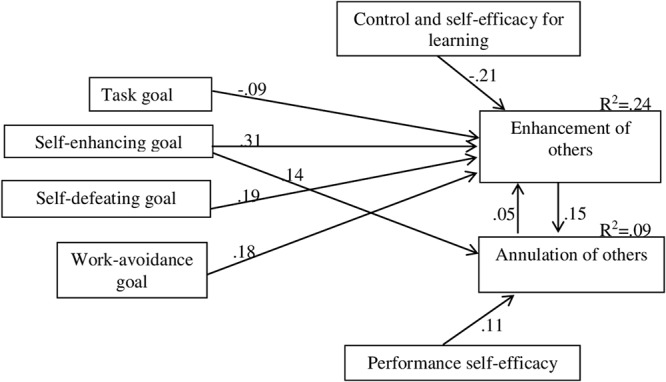
Explanatory model of the relations between goals, control and self-efficacy for learning, performance self-efficacy, and strategies of enhancement and annulation.

To assess the relationship between the different types of variables studied was specified a path diagram for the analysis of structural equation modeling in AMOS 22. The results suggest that, once the modification fit indices have been taken into consideration, the fit to the data of the model in Figure 1 (general model) is acceptable [χ^2^(8) = 5.204, *p* = 0.736] and that the fit statistics provide corroborating evidence (Table 5).

The results obtained for the general model show significant relationship from the four academic goals and control and self-efficacy for learning toward the strategy of enhancement of others. These relations are negative in the case of task goal and control and self-efficacy for learning. However, the strategy of annulation of others was only related with the ego self-enhancing goal and performance self-efficacy, in both cases in a positive way. The highest relationship in the model is that from the goal of ego self-enhancing toward the strategy of enhancement of others.

Then was confirmed that the general model’s fit of the data was acceptable by applying it to both groups of gender at once [χ^2^(8) = 10.747, *p* = 0.216] and the fit statistics provided corroborating evidence in both cases (Table 5). In addition, the same thing happened when was applied the model to the three clusters [χ^2^(12) = 18.861, *p* = 0.092].

### A Model About the Relationships With the Strategies of Deception and Comparison, Considering Gender and Groups With Different Motivational Characteristics

**Figure 2:**
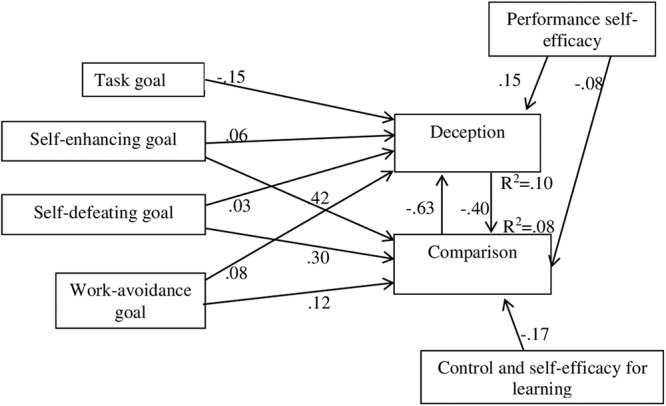
Explanatory model of the relations between goals, control and self-efficacy for learning, performance self-efficacy, and strategies of deception and comparison.

To assess the relationship between the different types of variables studied, were conducted analysis through structural equation modeling. The results suggest that, once the modification fit indices have been taken into consideration, the fit to the data of the model in Figure 2 (general model) is acceptable [χ^2^(4) = 3.228, *p* = 0.520] and that the fit statistics provide corroborating evidence (Table 5).

**Table 5:** Results of model fit and of the model’s application to genders and clusters (sample 1 and sample 2).

	Chi-	DF	Probability	Chi-	GFI	AGFI	IFI	TLI	CFI	RMSEA
	square		level	square/DF						
General model	5.204	8	0.736	0.651	0.998	0.990	1.005	1.018	1.000	0.000
Boys/girls	10.747	8	0.216	1.343	0.996	0.960	0.996	0.966	0.995	0.024
Clusters	9.219	8	0.417	1.571	0.997	0.970	1.000	0.994	0.999	0.005
General model	3.228	4	0.520	1.807	0.999	0.992	1.001	1.006	1.000	0.000
Boys/girls	2.448	4	0.654	0.612	0.999	0.988	1.002	1.024	1.000	0.000
Clusters	350	3	0.950	0.117	1.000	0.997	1.006	1.201	1.000	0.000

The model shows a similar pattern of positive relationships toward the two self-motivation strategies. These relations are from the two ego-oriented goals and from the goal of avoidance of work; the only difference seems to consist of these relations being more intense in the case of the comparison strategy. However, this pattern is broken by the relation of the task goal toward the strategy of deception, which is also negative. With regard to control and self-efficacy beliefs variables, the comparison strategy shows negative relationships with both variables and the deception strategy is positively related with performance self-efficacy.

Then was confirmed that the general model’s fit of the data was acceptable by applying it to both groups of gender at once [χ^2^(8) = 8.807, *p* = 0.359] and the fit statistics provided corroborating evidence in both cases (Table 5). In addition, the same thing happened when was applied the model to the three clusters [χ^2^(3) = 350, *p* = 0.950].

## Conclusion and Discussion

In brief, with respect to the first sample, can be highlighted that enhancement and annulation of others strategies positively correlate and that both are the variables with the lowest mean scores. It can be observed, however, that they exhibit a very different type of relation. The strategy of enhancing others is related with the four types of goals, whereas the strategy of annulling others is related only with the self-enhancing goal. In addition, in the first strategy, the relationships with the task goal and with the beliefs on control and self-efficacy for learning are negative – that is, there is a negative relationship with variables relating to the learning process. On the other hand, in the second strategy the relationship of the ego self-enhancing goal and the performance self-efficacy goal is positive – that is, there is a positive relationship with the variables relating to performance. Therefore, both strategies may respond to different patterns, such that the strategy of enhancing others is associated with deficiencies in the learning process and the strategy of annulling others is associated with processes in which consideration of performance predominates.

However, because this part of the study considers only two strategies related to the use of classmates, it does not allow us to ascertain if this possible pattern in the use of the two strategies is something that can occur with other kinds of strategies that also relate to the use of classmates. For this reason, it was considered that this study was insufficient and that it was necessary to use a new sample that allows first whether this pattern is repeated with other strategies to be ascertained and second whether results about the other variables are corroborated.

With the second sample, once again was found that both strategies positively correlate with each other and that both are the variables with the lowest mean scores. Also was found that the deception and comparison strategies were explained by all the goals, except for the task goal with regard to the strategy of comparison. And was found that the strategy of comparison was characterized by a negative relationship with beliefs. On the other hand, the strategy of deception only showed a negative relation with the task goal, and there was a positive relation with performance self-efficacy.

Therefore, it appears that the strategy of deception is more related to the beliefs of the student regarding their performance and to the lack of the pursuit of learning as an end in itself, with the relations of the rest of the goals being very low. This is contrary to what occurs with the strategy of comparison, which seems more related with the goals of ego orientation and effort avoidance and is characterized by low beliefs relating to learning.

The management of one’s own motivation is another element of what has been defined as SRL. To achieve it, the student has multiple strategies that can be used for self-motivation during their learning. In this paper, were addressed four specific motivation strategies, whose common element is a use of peers as a reference (enhancement and annulation of others, deception and comparison).

The mean scores obtained on the employment of these four strategies of self-motivation were of a low to medium level, which was predictable (Suárez and Fernández, 2011b). In any case, was observed a higher use of the strategy of annulation of others relative to the rest of the strategies. Furthermore, the four strategies seem to show a same pattern, in which both pairs correlate positively and significantly with one another.

The results obtained with the first sample regarding different patterns in both strategies, with the result that the strategy of enhancing others was associated with shortcomings in the learning process and the strategy of annulment of others was associated with processes in which the consideration of performance predominated, were not confirmed with the second sample. Nevertheless, the strategies of enhancement of others and deception seem to show a more disadaptative type of motivation because they correlate negatively with the task goal, in which the pursuit of learning is an end in itself (Skaalvik, 1997; Nietfeld et al., 2014), and with the control and self-efficacy beliefs, at the same time as correlating positively with the work-avoidance goal. Conversely, the strategy of annulation of others is the only one that fits an adaptive pattern of learning, since in the cluster analysis was observed that the students that report on its use most were the only ones that reported a greater use of both the task goal and the ego self-enhancing goal, which may allow them to direct their study toward both the objective of learning as an end in itself and the pursuit of academic performance. That is, the results indicate that the group of students with the highest level for the strategy of annulation is the one that seeks to the greatest extent to learn, that increasingly seeks to obtain good grades and that presented the highest levels for beliefs about their ability to learn and perform. However, it must be remembered that these results refer only to the strategies studied here. Therefore, there are probably other self-motivation strategies that not only are more frequently used but that also have more adaptive potential. In any case, it remains curious that in studying the strategies that have peer relations as a common element it emerges that annulation of others is the one that is most adaptive for learning. This may reflect the inadequacy of the use of external elements in order to modify internal aspects such as academic motivation.

On the other hand, it is also important to note that the worst motivational levels were obtained by the two groups of students with the lowest levels in the use of self-motivation strategies. The students with the lowest levels in the use of self-motivation strategies are those who to the least extent seek to learn and perform, and they exhibit the lowest levels in beliefs about their own ability to learn and perform. It is not surprising that these students report the lowest use of self-motivation strategies, because they are the students with the highest levels of demotivation. And it is reasonable to suppose that the lower interest that they have in their studies brings with it a lower interest in applying self-motivation strategies.

With regard to gender, it should be indicated that only were found differences in the use of the strategy of deception. And this occurred despite what we might have foreseen at the outset, as it is usual for girls to show significant and higher scores in certain variables that involve a social component (e.g., Trucco et al., 2013; Petersen and Hyde, 2014). Only was found that boys use the strategy of deception to a greater extent. It is logical since they are also the ones who reported to a greater extent an orientation toward the goal of ego self-enhancing and boys are more likely than girls to lie when interactions are fully anonymous and deceptive messages can secure a benefit (Dreber and Johannesson, 2008). Furthermore, in both samples girls scored more highly in the ego self-defeating goal, whereas boys scored more highly in the work-avoidance goal.

Finally, were obtained different models on relationships between the variables of academic goals, self-efficacy and self-motivation strategies. These models were also confirmed with both genders. And there was also confirmation with the groups of students created based on their motivational characteristics.

In terms of limitations to the study, alternatives models were not considered. They should be considered in future research, in the same way that other variable into the model. So it is important to highlight the need to study the diversity of self-motivational strategies. Moreover, it is important to highlight the need to use other types of instruments too. Finally, it would be of interest to apply other designs. The results obtained do not demonstrate causality, hence the need to use other methodologies. Moreover, a longitudinal design would allow us to study the evolution of these strategies.

By way of conclusion, it is worth highlighting the need for further research on self-motivation strategies, so that this line of research takes into account not only self-regulation of the cognitive and behavioral components of learning but also the self-regulation of the student’s own motivation (Zimmerman, 2013). More specifically, the strategies of enhancement of others, annulation of others, deception and comparison have a special relevance because they situate the student in a social, interactive context, highlighting the importance of considering the approach of situated learning at the same time as students’ personal characteristics and thoughts (Dermitzaki and Efklides, 2001).

Educational and practical implications of the work are related mainly to the tutorial work of the teachers. SRL is a key competency that students should learn (Steinbach and Stoeger, 2018) so educators should know SRL procedures in order to show their students to use these techniques and thus impact on students’ learning and achievement. In this way, teachers could train their students in the use of adaptative motivational self-regulation strategies, while discarding those other inadequate strategies, in order to improve their motivation and learning outcomes.

## Author Contributions

JS, AF, and ÁZ conceived and designed the study, organized the database, performed the statistical analysis, and wrote the sections and first draft of the manuscript. All authors contributed to manuscript revision and read and approved the submitted version.

## Conflict of Interest Statement

The authors declare that the research was conducted in the absence of any commercial or financial relationships that could be construed as a potential conflict of interest.
